# Supplementary material for: A doublecortin-domain protein of Toxoplasma and its orthologues bind to and modify the structure and organization of tubulin polymers
Source: BMC Mol Cell Biol. 2020 Feb 28;21:8. doi: 10.1186/s12860-020-0249-5 (PMC7048138; doi:10.1186/s12860-020-0249-5)
Supplement: Supplementary file 1 — Additional file 1: Table S1. Data Collection, Phasing, Ramachandran Statistics, and Refinement Statistics for TogoA.17199.a.B3.PW38274. Table S2. Oligonucleotides and gBlock fragments used in this study. [file 12860_2020_249_MOESM1_ESM.pdf]

Table S1: Data Collection, Phasing, and Refinement Statistics for TogoA.17199.a.B3.PW38274

**Parameters**	**Iodide**	**Native**
Wavelength (Å)	1.5418	1.5418
Space Group	$P2_12_12_1$	$P2_12_12_1$
Cell Dimensions		
a, b, c (Å)	36.40, 53.28, 96.25	36.86, 53.23, 96.38
α, β, γ (°)	90.000, 90.000, 90.000	90.000, 90.000, 90.000
Resolution (Å)	50.00-2.00	50.00-2.00
R_{merge} (%)	4.5 (14.4)	5.9 (55.7)
$I/\sigma I$	44.1 (14.2)	18.0 (2.7)
Completeness (%)	98.7 (88.5)	99.8 (99.8)
Redundancy	14.0 (8.5)	6.1 (3.9)
Refinement		
Resolution (Å)		35.7-2.0
Reflections (#)		13372
$R_{\text{work}}/R_{\text{free}}$ (%)		18.6/26.1
MolProbity Score		1.3
Ramachandran Favored		97.8%
Ramachandran Allowed		2.2%
Ramachandran Outliers		0.0%
Number of Non-Hydrogen Atoms		
Protein		1463
Formate		6
Water		104
R_{msds}		
Bond Lengths (Å)		0.007
Bond Angle (°)		0.784
Average B Factors (Å^2)		
Overall		45.67
Protein		45.59
Formate		55.3
Water		45.29

Table S2. Oligonucleotides and gblock fragments used in this study

name	sequence
S1	GGAGGAACAGGATCTGGTGGAACTGGTTCTGGTGGTTCAGTGAGCAAGGGCGAG
AS1	GCAGCTTCTGTTTACTTAAGCTACTTGTACAGCTCGTCC
CvDCX1_<br>gBlock	gaattccctttttagatccgctagcaaaATGGAGTATTTGCGATACGATGAGAGGCCGGCGGCA<br>GTGCCGGGTCCCTCTACCAACGCAATACAGAACCCGTACAACACAAACCACGTGATGCAGGGA<br>GAGTACGGGTTTGAAGAGCCCTCTGTATGGGAGAGGCTCACAGATCCGTCCAGGTATACAGGG<br>GTGCACAGAGAGCGATTTGATGAGTTTGGTCGTGGGCGAGGTCTAGCAGGGCGGGAGAACGTG<br>TACTACTTCGATGGGATGACGGAGAGTCCCTCGAGGTGTCACGAGATCTACTCGACCGTGGTC<br>ACACAAAAACGAAAGGCCGCTGTCACACCAGGAACCCTGGGGGTTCAAAAGTTCGGAACCCAG<br>GCAGTGACCCCCAAGTTGATCTGGGTGTACCGAAACGGTGACAGGTCGCACGAGGGTCACCCC<br>GTCTACCTGCGAAATTCCATCAAGACGATGGAGTTCCTCTACAGAGAGTGCACCAAGGTGGCC<br>TGTCCGTTCACGGGCCCTGTTTTGAAGATTTACGACCAGAATCTCAAGAGAGTGAAGAAGTTG<br>GAGCACTTCGTTGACGGGGGGAAGTACCTATGCTGCGGGGGGGAGCTCCCCTCTCTGGACAAA<br>CTCGAGAAGTTCCTCTCGAAGTTCGTCTTTGTCATCggaggaacaggatctg
CvDCX2_<br>gBlock	gaattccctttttagatccgctagcaaaATGCCCGACGCCTTCCTTGAGAGAATGACCGACGCC<br>TCCCAGTACACGGGAGCCCACAAAGCCCGCTTCGATGCTGACGGGAACGGCCGCGGTCTGGCC<br>GGGAGAGAGAACGTGGTGTACTACGATGGCTCCACAGAGTCCGCAACCCGCAGCCATGCCGTC<br>GAGAATACAGTGCAAAAGAAGGGAAATCGTAAAGCAGTTGTGACGGGCCCTCTTGGGTGCCAG<br>AAGTTCGGAACCCAGGCCGATACCCCCATTTCCTTCACAATCTACAAGAACGGCGACAAGTTC<br>CACAAAGGCCACAAGATCCTTCTCAAGAAGCACTACCGCAACATGCAGCAGCTTATTGACGAG<br>TGCAATAAGCACGCCCAGCCCCTCACCGGTCCCATCCGCCGCCTCTACCGCACGGACCTGCGA<br>ACGTGGGTGAAGGAGCTCCACGAGTTTGAGGATGGCGCCAAGTACCTGTGCGTTGCCGGAGAG<br>CACCCGAAGGATGACATCGAGAAGATCCCCCCCGGGTTCTGGGAGggaggaacaggatctg
CvDCX3_<br>gBlock	gaattccctttttagatccgctagcaaaATGCCCAACGACGCCACCCTTGCAAGACTTACTGAC<br>ACTTCCAAGTACACGGGAGCCCACAAGCAACGTTTTGATGAGGACGGCAAAGGACGCGGTCTG<br>GCAGGGAGGGAGAACGTCACACACCACGACGGTTCCACTGAGTCTGCTGTTCGATCACACGCA<br>ATTGAGAAGACAGTGGACGAGAAAGCACACAAGAAATCAGTTGTGAAGGGCCCCCTTGGACAA<br>CAGAAGTTCGGCACCCAGGCCGACACCCCGATCTCCTTCATGATTTACAAGAACGGGGACAAG<br>AACCACAAGGGCCACAAGGTCCTTCTCAAGAAGCACTTCCGCAACATGCAGCAGTTGACTGAT<br>GAGTGCAACAAGCACGCGGCGCCTCTGACCGGGCCTATCCGACGTTTCTACCGTCCTGACCTG<br>AAGACCTGGGTTAAGGACCTGACGGAGTTCGAGGACGGCGGCAAGTACCTGTGCGTTGCCGGG<br>GAGAACCCGAAGGACGACATTGAGAAGATCCCCCCCGGGTTTTTCGAAggaggaacaggatct<br>g

name	sequence
VbDCX1_<br>gBlock	gaattcccttttagatccgctagcaaaATGGCACTTTCAAGCGCTGTCGGCCGCCACCGCAAC<br>ATATTCGAGAAACTGTGTGATCCCTCCCTCTATACAGGAGCACATAGGGAGCGATTCGATGAG<br>AACGGCAAGGGCAGAGGGCTGGCGGGACGAGAATACGTGTATTACGTTGATGGGATGACTGAG<br>AGCCCCACGCGATGCCACGAGGTATACTCGTCTGTGAGGGAGAGACCCAAACGCAAACCGCTC<br>AGAAGACCTCCCGCGCTCAAAGAGCGTATGTCCGGGGCACCGCCGAGGGCGAAGCTCATATGG<br>CTGTACAGAAACGGAGACAAGAAACACCTGGGCTCTCCCTCCTTCGTGCGGCCGCATGTGCAG<br>TCGCTGGACCTGCTGTATCGTGAAGCGTCAGCTGAGATCCGCCTGCTCACCGGGCCGGTCAAA<br>CGTCTGTATGACCAACATCTGGAGGCAATCAGAGACATAGACGACCTCACCGACGGCGGCAAG<br>TATCTCTGCTGCGCCGGCGAGCAGCCATGTATGGAGAAGCTGGACCATTTCCTCTCTGACTAC<br>GTCGTATGGCCGACTCTGCTGCACACGAGAggaggaacaggatctg
VbDCX2_<br>gBlock	gaattcccttttagatccgctagcaaaATGTCGGGAAGGAAGAGCCCCGAGAAGCAGAGCATC<br>TTCGACAGGCTCACCGACCACACCCAATACACGGGTGCCCATAAGCACCGTTTCGACGAGGGC<br>GGTAAGGGGCGAGGGATTGCTGGTCGGGAGGAGCTGGTCAACATTGACGGCTCCACCGAATCT<br>TCTGCCCGCCGCCACGCCGTGGAGAAGACAGTCGACCATGTCGAGCGTGCCGGGCGCAAGCCA<br>GTGGTGCAGGGCGCCCTTGGCCAGCAGAAGTTCGGCACCCAAGCTGAGACACCCATCACCATT<br>TGGCTCTACAGGAACGGTGACAAGCACTTCAAGGGCGTCAAGTTCATCGTCAAGAAGACCATC<br>CGGAACGTGGAACAGTTCGTTGCTGAGGCGGGCAAGTCGGGCTGCAGCCCCAAGAGCGGGGTG<br>ATCCGCAAGATCTACAAGCAGAACATGAAGACCATCATCAAGGACATCACCGAATTCGAAGAC<br>GGCGAGAAGTACCTCTGCTGCGGAGCCGAGAAGCCCCAGGATGACCCCGAGCACATCCCTGCT<br>GCCTTCCTCGAGggaggaacaggatctg
VbDCX3_<br>gBlock	gaattcccttttagatccgctagcaaaATGGCGCCGAAGCAAAACATCTTTGACAAGCTCACC<br>GATAGCTCCCAATACACGGGGGCCCACAAGCACCGCTTCGACGACTCCGGCCAGGGTCGTGGC<br>ATCGCTGGGAGAGAGGAAATCGTCAACGTGGATGGTTCAACCGAGTCGACCGCACGCAAACAT<br>GCCGTGGAGAAGACCGTCGACCACGCCGAGCGTGCGGGTCGTAAGCCAGTAGTGCAGGGGCCC<br>CTCGGCCAGCAGAAGTTCGGGACTCAGGCGGAGACACCCATCTCCATCTGGCTGTACAAGAAC<br>GGCGACAAGCACTTCAAGGGAGTCAAGTTCGTCGTCAAGAAGACCATCAGAAACATGGACCAG<br>TTCATCGCTGAGGCCAACAAGCAGGGCTGCCAGCCCAAGAGCGGTGTGATTCGTAAGATCTAC<br>AAGCAGAACATGAAGACTGTTGTCAAGGACCTGGCAGACTTCGAAGACGGCGAAAAATACCTC<br>TGCTGTGGACCCGAGAAGCCCCAGGAGggaggaacaggatctg
S2	GAATTCCCTTTTAGATCCGCTAGCAAAATGGAGAATTTTGATGAAGTTATAAAAG
AS2	ATGGATGCTTACAAGAATTTATCTTGTGGGTCTTTTCTT
S3	AAGAAAAGACCCACAAGATAAATTCTTGTAAGCATCCAT
AS3	CAGATCCTGTTCCTCCAGTTAGAAAGTGAAGACTCAAG
S4	GAATTCCCTTTTAGATCCGCTAGCAAAATGGCGAGTAAGAAATCTCAAGA
AS4	CAGATCCTGTTCCTCCTTGGAACAGAGATGGCG
S5	GGTTCCGGTGGGTCAATTCCAGCTCCTAGATTAATGTG
AS5	GGGCAGCTTCTGTTTACTTAAGTCACTGAATCACCCATTCGC
S6	GGTTCCGGTGGGTCAAATGTTTTTGAACGGCTAACC

name	sequence
AS6	GGGCAGCTTCTGTTTACTTAAGTCACTGAATCACCCATTCGC
S7	GAATTCCCTTTTAGATCCGCTAGCAAAATGGTGAGCAAGGGC
AS7	TGACCCACCGGAACCAGTTCCACCAGACCCGGTACCTCCCTTGTACAGCTCGTCCA
S8	GTACAAGTCCGGACTCAGATCTATTCCAGCTCCTAGATTAATGTG
AS8	GGATCCCGGGCCCGCTTAAGTTATCACTGAATCACCCATTCGC
S9	gtacaagtccggactcagatctAATGTTTTTGAACGGCTAACC
AS9	ggatcccgggcccgcttaagttaTCACTGAATCACCCATTCGC
S10	AATTCTGCAGTCGACGCTTAAGCGGGC
AS10	CCGGGCCCGCTTAAGCGTCGACTGCAG
S11	CCCAAGCTGGCTAGCGTTTAAAGTTAAGC
AS11	AACGGGCCCTTAAGACTCGAG
S12	GTACAAGTCCGGACTCAGATCTGAGTATTTGCGATACGATGAGAGG
AS12	GGATCCCGGGCCCGCTTAAGTTAGATGACAAAGACGAACTTCG
S13	GTACAAGTCCGGACTCAGATCTCCCGACGCCTTCCTTG
AS13	GGATCCCGGGCCCGCTTAAGTTACTCCCAGAACCCGGGGGGG
S14	GTACAAGTCCGGACTCAGATCTCCAACGACGCCACCCTTGC
AS14	GGATCCCGGGCCCGCTTAAGTTATTCGAAAAACCCGGGGGG
S15	GTACAAGTCCGGACTCAGATCTGCGCCGAAGCAAAACATCT
AS15	GGATCCCGGGCCCGCTTAAGTTACTCCTGGGGCTTCTCGG
S16	GTACAAGTCCGGACTCAGATCTGCACTTTCAAGCGCTGTC
AS16	GGATCCCGGGCCCGCTTAAGTTATCTCGTGTGCAGCAGAGTC
S17	GTACAAGTCCGGACTCAGATCTTCGGGAAGGAAGAGCCC
AS17	GGATCCCGGGCCCGCTTAAGTTACTCGAGGAAGGCAGCAGG
S18	GTACAAGTCCGGACTCAGATCTGAGAATTTTGATGAAGTTATAAAAGAATATCAG
AS18	GGATCCCGGGCCCGCTTAAGTTAAGTTAGAAAGTGAAGACTCAAGTTTCG
S19	ATCGAGATCTATGGCGACACGACAGGCAG
AS19	GCTACTTAAGTTACACAACGAAGAACTGGCTC
S20	GTACAAGTCCGGACTCAGATCTGCGTGCGGCATCCCATGG

name	sequence
AS20	GGATCCCGGGCCCGCTTAAGTTACACAACGAAGAACTGGCTCG
TgDCX gblock	ggttccggtgggtcagcgtgcggcatcccatggaagcttgctcgacgggatgaactaatggcgacacgacaggcagaaaggccgggcgagtactttcctccgccctatccgccgtgtccgcctacggttgtcacgccgcttcggacaagtgcatacgatttccctgaagccaccttcgtcacgaggccatgccttcccgccaagaaggcgacaggacacaaaaatgtttttgaacggctaaccgacacggcctactacacgggatcgcaccgagaacggtttgacgagttcggaaatggtagaggaattgctggtagagaatacttatatgcctacgacggattgacggagtctccaagtagatgccacgaagtctactcctctgtcattaagcgacctcgaaaacccgttgtcacccccggtaccttgggtatccagcggttcggagttcaaattccagctcctagattaatgtggctttaccgcaacggcgataaacacgacgacggcactcctttcttcgtccgcccctacatcaaatccatggagtctctttatcaacagatcactaaggaaataactcccatcgctggaccggtccgaagaatcttcgaccagaatttccgtgtcatcaccgatcttgatgacattgttgatggcgcgaaatacttgtgtacctctggagaaccacctgcagcttacgatcgactcgagaaatttctgagcgaatgggtgattcagaagtcgcaaacaaaggttccgagccagttcttcgttgtgtagcttaagtaaacagaagctgccc
TgDCX-R152I-D201R gblock	ggttccggtgggtcagcgtgcggcatcccatggaagcttgctcgacgggatgaactaatggcgacacgacaggcagaaaggccgggcgagtactttcctccgccctatccgccgtgtccgcctacggttgtcacgccgcttcggacaagtgcatacgatttccctgaagccaccttcgtcacgaggccatgccttcccgccaagaaggcgacaggacacaaaaatgtttttgaacggctaaccgacacggcctactacacgggatcgcaccgagaacggtttgacgagttcggaaatggtagaggaattgctggtagagaatacttatatgcctacgacggattgacggagtctccaagtagatgccacgaagtctactcctctgtcattaagcgacctcgaaaacccgttgtcacccccggtaccttgggtatccagcggttcggagttcaaattccagctcctaTTttaatgtggctttaccgcaacggcgataaacacgacgacggcactcctttcttcgtccgcccctacatcaaatccatggagtctctttatcaacagatcactaaggaaataactcccatcgctggaccggtccgaagaatcttcCGccagaatttccgtgtcatcaccgatcttgatgacattgttgatggcgcgaaatacttgtgtacctctggagaaccacctgcagcttacgatcgactcgagaaatttctgagcgaatgggtgattcagaagtcgcaaacaaaggttccgagccagttcttcgttgtgtagcttaagtaaacagaagctgccc
S21	GTACAAGTCCGGACTCAGATCTAGTCGAGCATACGCGGAC
AS21	GGATCCCGGGCCCGCTTAAGTTATCAGTCTCGAAGAGACCAGAAATCTGC
S22	GTGAACCGTCAGATCCGCTAGCGTTTAAAGTTAAGCTTGCCACCATGGTGAGCAAGGGCG
AS22	AGATCTGAGTCCGGACTTGTACAGCTCGTCCATGC
Cvel_18664_I85R-R134D	ggttccggtgggtcaCCCGACGCCTTCCTTGAGAGAATGACCGACGCCTCCCAGTACACGGGAGCCCACAAAGCCCGCTTCGATGCTGACGGGAACGGCCGCGGTCTGGCCGGGAGAGAGAACGTGGTGTACTACGATGGCTCCACAGAGTCCGCAACCCGCAGCCATGCCGTCGAGAATACAGTGCAAAAGAAGGGAAATCGTAAAGCAGTTGTGACGGGCCCTCTTGGGTGCCAGAAGTTCGGAACCCAGGCCGATACCCCCAgaTCCTTCACAATCTACAAGAACGGCGACAAGTTCCACAAAGGCCACAAGATCCTTCTCAAGAAGCACTACCGCAACATGCAGCAGCTTATTGACGAGTGCAATAAGCACGCCCAGCCCCTCACCGGTCCCATCCGCCGCCTCTACgaCACGGACCTGCGAACGTGGGTGAAGGAGCTCCACGAGTTTGAGGATGGCGCCAAGTACCTGTGCGTTGCCGGAGAGCACCCGAAGGATGACATCGAGAAGATCCCCCCCGGGTTCTGGGAGtagcttaagtaaacagaagctgccc
S23	GAATTCCCTTTTAGATCCGCTAGCAAAATGAGTCGAGCATACGCGGAC

name	sequence
AS23	CAGATCCTGTTCCTCCGTCTCGAAGAGACCAGAAATCTGC
AS24	GCTACTTAAGTTAGACAACGGGTTTTCGAGGTC
S24	ATCGAGATCTACCCCCGGTACCTTGGGTATC
TgGT1_ 266630 (“CPH1”) gblock	ggttccggtgggtcaAGTCGAGCATACGCGGACTTGGTTGTTTCGCGGAAGGCGAAGGCCCCGCCTTTCTCGGTGGATAACAACGATGGGCGGCCAAAACGTCCAAATCGGGTTTGGTTCGACAGAGACAGGGCAAAAGCGTGCAACGATATGAGACGAGAATATCCTCTCCACTACTACATTTCGTCCTACGACGTCTACGCGTTTCTTGCCACCCTTAGGCAGGGTGCAGATCCTGCGAAAAGAGATGCTGCTGGACGCACCCCTCTCGACCTTGCTGTGCAGATGGCAGTGGAGCTAATTGAGACGTCGCTCTGCACGTCTTTCCCTATCGACAATATCGACGTTACTCCGGCGGCAACTTTGAAGCCAGCGAAAGACTCTCCGCCAGAAAGTCGTCCGGAGACTTTCACCAATCCCTTTCGGGTAAAGTACCGCGACGCGAACGCCAGCATGTATGTTCAGCACGATATCATGCGGTATCGCTGCGCAGACTCGGATGTCTACAAATCCTGTCGGGAGCCGCCATTCCCTCTTCCCTCAGATGGTCACGATGGTGCAGAACGGACTTACCAGGTGGCCACCTTTCCACCACAAGAGTTTCGCATAAAAAACGGCGACTCTGAAGATTTCTTCAGGGACCAACAGCGCGCGCGACGAACACAATCTCCATCGTTTCCTCTTCCTCGGGGCGACGTCAGTCGCTACTACGCAGCTGCTCACAATTTGCGCCCGCAACAGTTCGCTTACGCCTATGATGCTAACGGCAAGCCGATGTCGTGGTCTCGCCTTCAGCGCGAGCAAGTTCAGTTGGAGCAGGAACACCTACAGGGAGAGTTTTTGGTGGCTGTGGAGCAGTTTCTCGAGCGTCCGCCGTCGAAGGCCGCGATGTCTCAGATGCGCGAGCTGATACGGCGGCTCAACCTCATGATGGTTATCATCAAGAAGTATGAACTCTGTCTGCCGCTGAAGCGTGAAGCAGCAGAAAAGGCGAAGGCTTACATCGGTACGGCGCTCACGTACCCGTACCTCTACACGGCGTCCATGTACGAGGCGTTCAAGCGCTACCCCAGAACGCCTGCGGGGCAGGCATGGGACGCTCTTAACCCTGCAGACAGCCGCCGGCTGGTGTTGATTATCTTGTCGCTGAAGATGCACGGCCATGAACTATTCTCGTTTATGGGCACCGTTTGCCGGCTGTTCAACTCACTTATGGAGCAGCTCGGTCTCTGCGATGACCGCTGTGGCACTGGTGGACATTGGTGTAAGCTGGGCAGTGAGTGCAGTCAGTTCTACCTTCACATGGCCTTTTCACTGGACGATGCTCAGCTCTTTCAAAAGTTCATCACGCAGCAGCAACTCTTCGACAAACAGGACATCTTCAAGTGGGAGGATCTCCTCTATACCCTCATGCAGCATCGCGAAAGGTTTAGGCCGTACTTCGCGGCTCTCTTGGCGAGGCGCCTCAAGGCGCGTGTGCAGAAGAGATTGATGAACTCGGCAGATCAGCAAGCCTACCTGCCAGAAACCGTCAAGTATCCCTGCGAGGAACGTTACTTCACCCGTCAGCTACTCGGTGAATATTCTGAAATTTTTGGAGAGTCGGAAGCAGAAGAATTCAACAGTATGCTGCGTCACTGGCGATACGGCGCAGCCCGGCAGGCACCTGGCACGGGCTCTGAGGAAGACAACATTCTCCCGGCTCCGCTACCTCCTGTTTCTGAGTCTCCGGCGGCAGATTTCTGGTCTCTTCGAGACTGActtaagtaaacagaagctgccc

name	sequence
DCX-DCX gBlock	GTACAAGTCCGGACTCAGATCTGCGTGCGGCATCCCATGGAAGCTTGCTCGAC<br>GGGATGAACTAATGGCGACACGACAGGCAGAAAGGCCGGGCGAGTACTTTCCT<br>CCGCCCTATCCGCCGTGTCCGCCTACGGTTGTCACGCCGCTTCGGACAAGTGC<br>ATACGATTTCCCTGAAGCCACCTTCGTCACGAGGCCATGCCTTCCCGGTGTCCA<br>GATCCCTGCACCACGCCTCATGTGGTTATATAGAAATGGGGACAAGCATGATGAT<br>GGGACACCATTTTTTGTGAGGCCGTATATTAAGAGCATGGAAAGTTTATACCAGC<br>AAATTACAAAAGAGATCACACCAATAGCAGGTCCCGTTAGACGCATTTTTGATCA<br>AAACTTTAGAGTTATTACAGACCTCGACGATATCGTAGACGGAGCAAAGTATTTAT<br>GCACGAGTGGTGAGCCTCCAGCTGCATATGACAGGTTGGAAAAGTTCTTATCTG<br>AGTGGGTTATACAAAAAAGTCAGACTAAACACGAAGTCTACTCCTCTGTCATTAA<br>GCGACCTCGAAAACCCGTTGTCACCCCCGGTACCTTGGGTATCCAGCGGTTCG<br>GAGTTCAAATTCCAGCTCCTAGATTAATGTGGCTTTACCGCAACGGCGATAAACA<br>CGACGACGGCACTCCTTTCTTCGTCCGCCCCTACATCAAATCCATGGAGTCTCT<br>TTATCAACAGATCACTAAGGAAATAACTCCCATCGCTGGACCGGTCCGAAGAATC<br>TTCGACCAGAATTTCCGTGTCATCACCGATCTTGATGACATTGTTGATGGCGCGA<br>AATACTTGTGTACCTCTGGAGAACCACCTGCAGCTTACGATCGACTCGAGAAATT<br>TCTGAGCGAATGGGTGATTCAGAAGTCGCAAACAAAGGTTCCGAGCCAGTTCTT<br>CGTTGTGTAACTTAAGCGGGCCCGGGATCC
P25-P25 gBlock	GTACAAGTCCGGACTCAGATCTGCGTGCGGCATCCCATGGAAGCTTGCTCGACGGGAT<br>GAACTAATGGCGACACGACAGGCAGAAAGGCCGGGCGAGTACTTTCCTCCGCCCTATC<br>CGCCGTGTCCGCCTACGGTTGTCACGCCGCTTCGGACAAGTGCATACGATTTCCCTGA<br>AGCCACCTTCGTCACGAGGCCATGCCTTCCCGCCAAGAAGGCGACAGGACACAAAAAT<br>GTTTTTGAACGGCTAACCGACACGGCCTACTACACGGGATCGCACCGAGAACGGTTTG<br>ACGAGTTCGGAAATGGTAGAGGAATTGCTGGTAGAGAATACTTATATGCCTACGACGGAT<br>TGACGGAGTCTCCAAGTAGATGCCACGAAGTCTACTCCTCTGTCATTAAGCGACCTCGA<br>AAACCCGTTGTCACCCCCGGTACCTTGGGTATCCAGCGGTTCGCTAAAAAGGCTACTGG<br>TCATAAGAACGTCTTCGAGAGATTGACAGATACAGCTTATTATACTGGGTCACATAGGGA<br>GCGGTTCGATGAATTTGGTAACGGCCGGGGTATCGCGGGACGTGAGTATCTTTACGCAT<br>ATGATGGCCTCACAGAAAGCCCTTCTCGGTGTGTTCCGAGCCAGTTCTTCGTTGTGTAA<br>CTTAAGCGGGCCCGGGATCC
