# Supplementary material for: A doublecortin-domain protein of Toxoplasma and its orthologues bind to and modify the structure and organization of tubulin polymers
Source: BMC Mol Cell Biol. 2020 Feb 28;21:8. doi: 10.1186/s12860-020-0249-5 (PMC7048138; doi:10.1186/s12860-020-0249-5)

Supplementary Figure 1

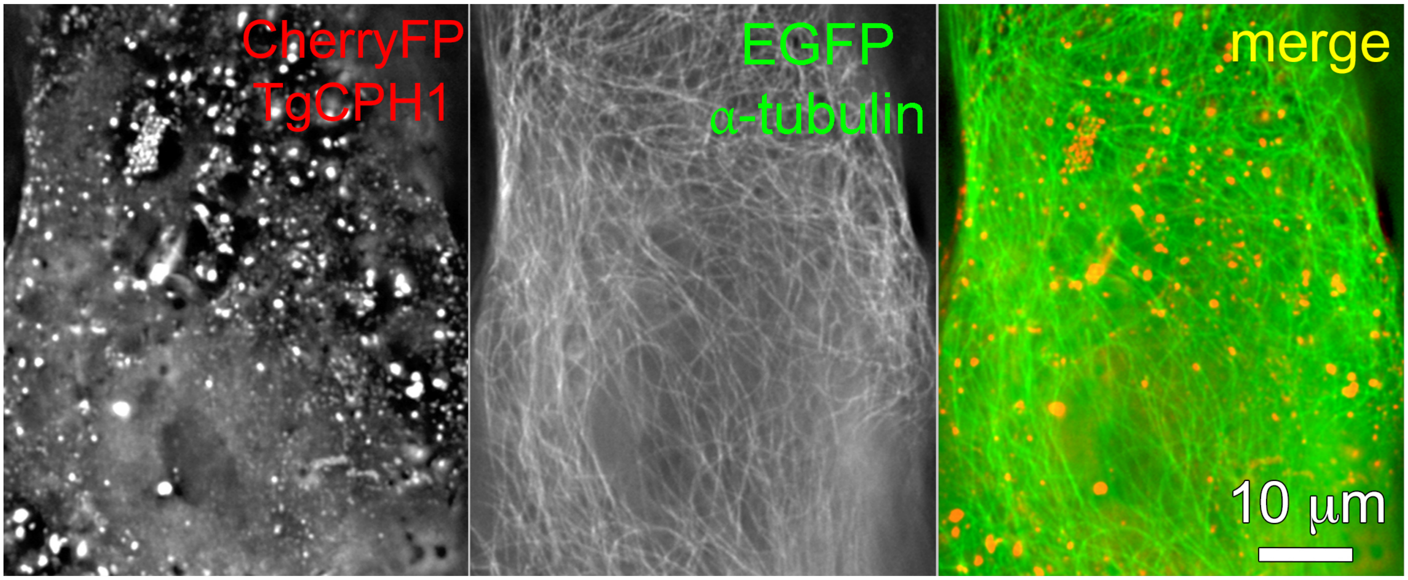

Supplementary Figure 2

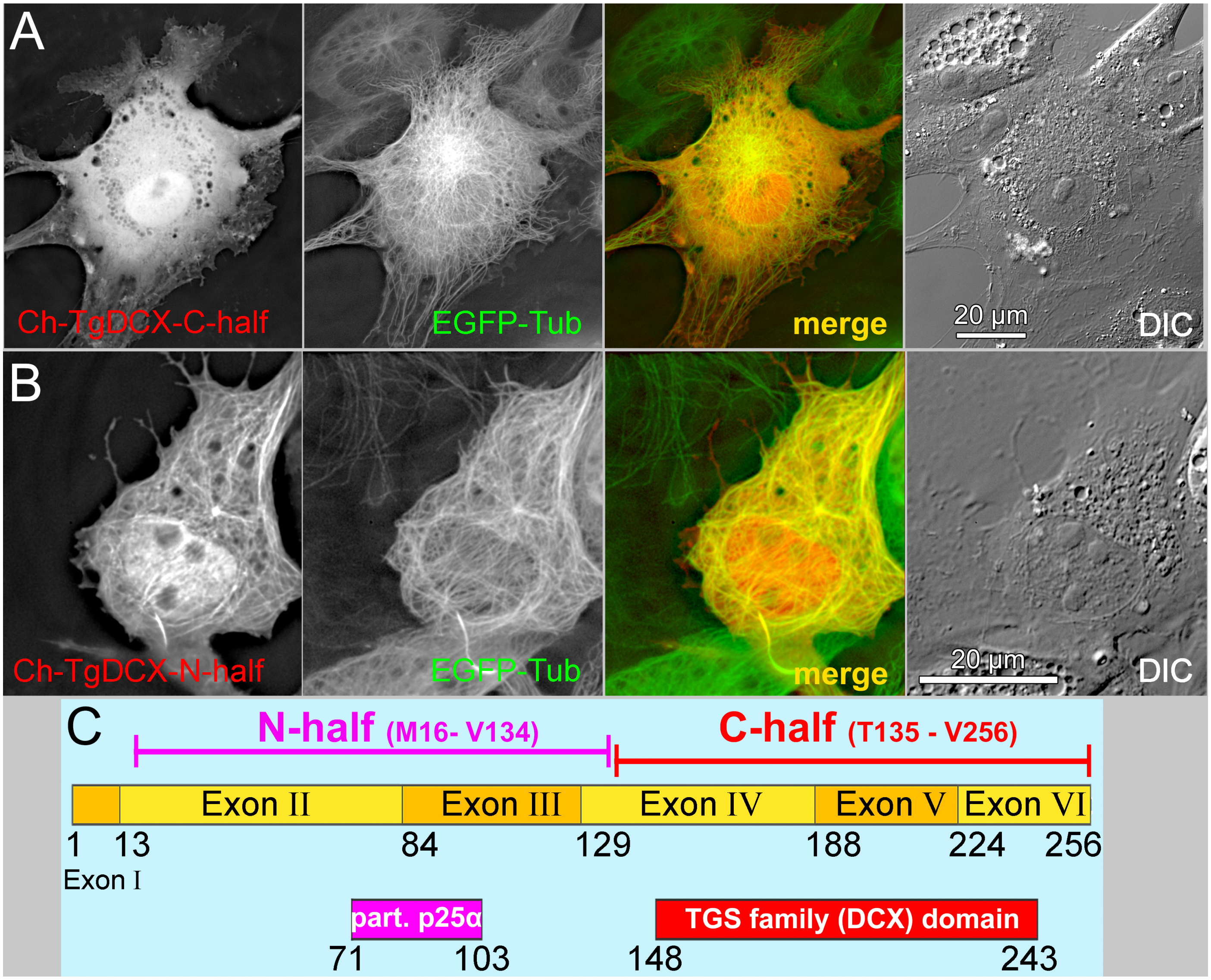

Supplementary Figure 3

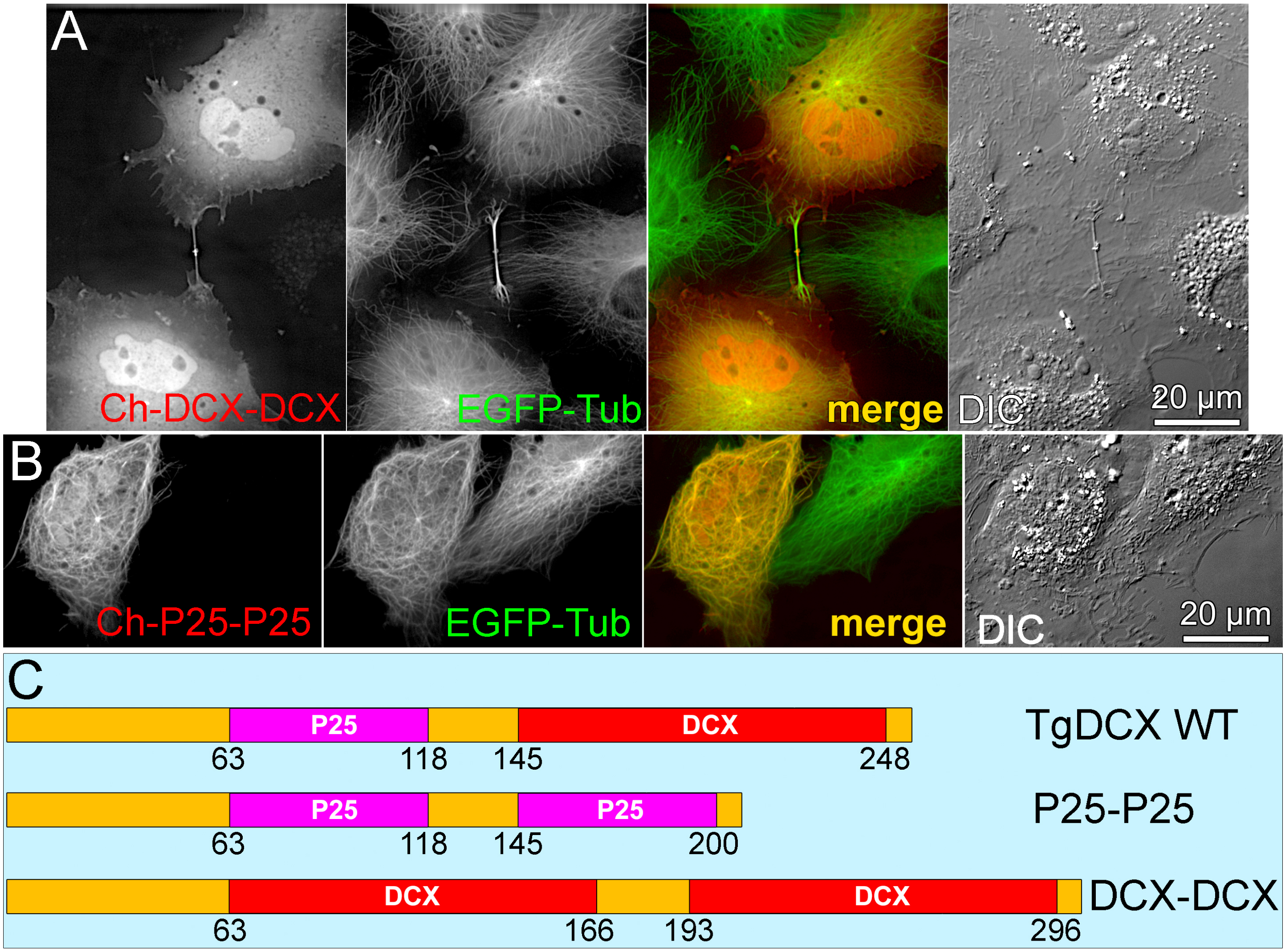

Supplementary Figure 4

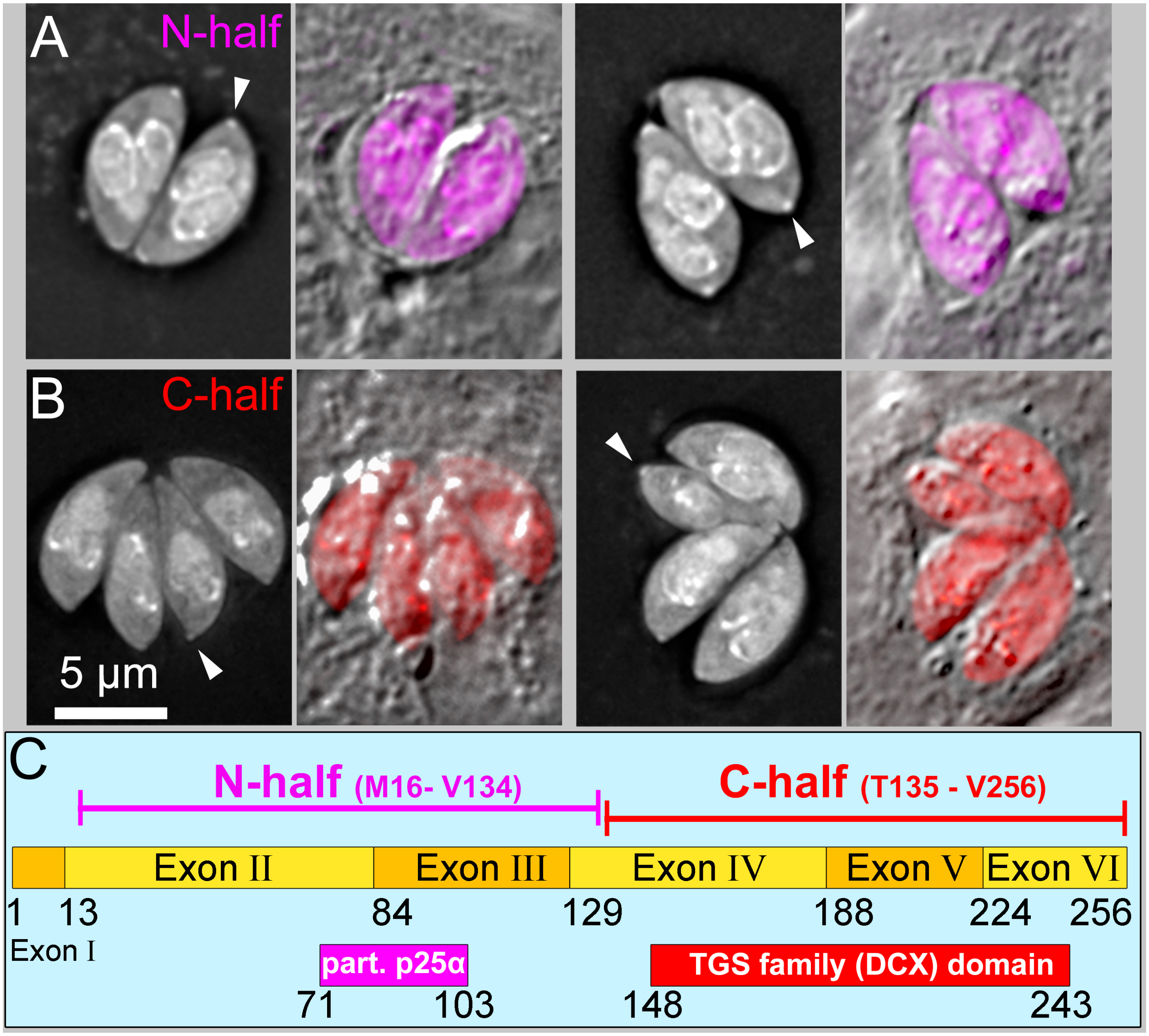

Supplementary Figure 5

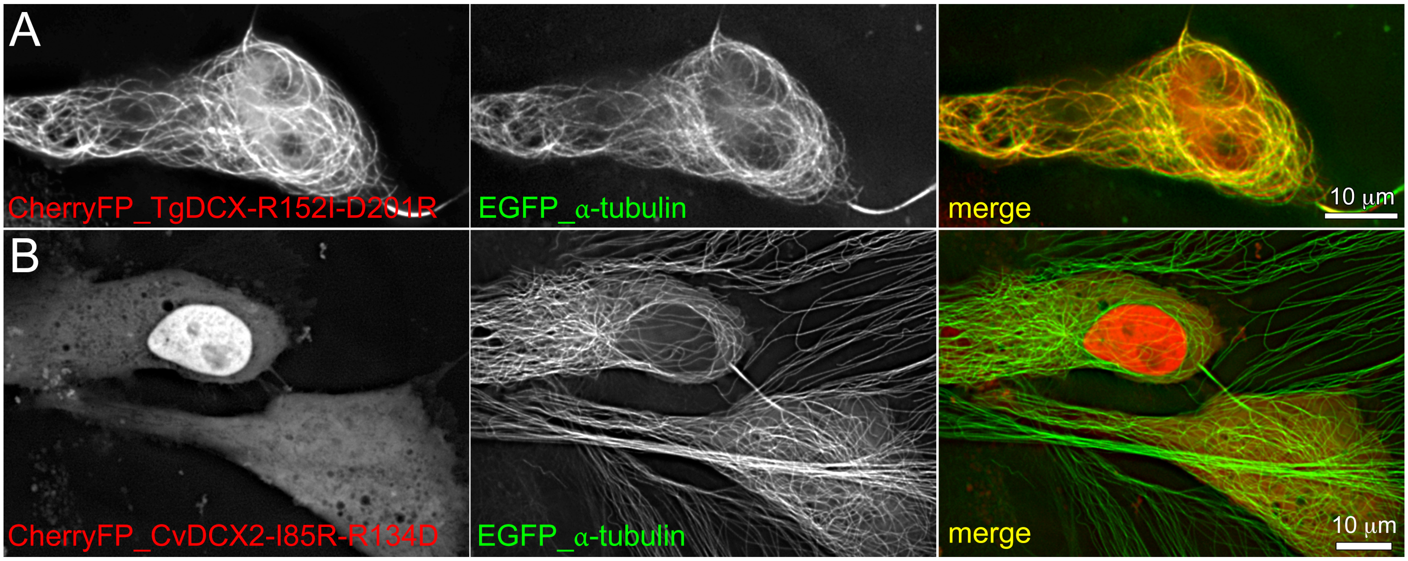

Supplement: Supplementary file 2 — Additional file 2: Figure S1. TgCPH1 does not bind to microtubules in the absence of TgDCX in Xenopus S3 cells. Deconvolved wide-field images of Xenopus S3 cells expressing mCherryFP-TgCPH1 (red), and EGFP-α-tubulin (green). When expressed alone, FP tagged CPH1 is diffusible cytoplasmic and in vesicles, not associated with microtubules. Figure S2. Effect of single domain constructs on microtubules in Xenopus S3 cells. A) TgDCX135–256, containing the C-terminal half of TgDCX, which includes the DCX domain, remains cytoplasmic, not binding to microtubules. B) TgDCX16–134, containing the N-terminal half of TgDCX, which includes the partial P25α domain, binds to microtubules in Xenopus S3 cells but does not modify MT architecture. C) Schematic showing the N-half and C-half fragments of TgDCX. Figure S3. Effect of domain swapping on TgDCX interaction with microtubules in Xenopus S3 cells. A) A construct with two copies of the DCX domain remains cytoplasmic, not binding to microtubules. B) A construct with two copies of the partial P25α domain binds to microtubules but does not change their architecture. C) Domain structure of the WT TgDCX and the domain-swapped constructs. The sequences outside the defined domains, shown as orange segments, are identical in all three molecules. Figure S4. Deconvolved wide-field images of parasites expressing either eGFP-TgDCX16–134 (“N-half”, A) or eGFP-TgDCX135–256 (“C-half”, B). Arrowheads indicate conoids. (C) Diagram of the domain structure of TgDCX. Numbers indicate amino acid residues. The full-length protein is 256 aa. Figure S5. Residues R152 and D201 in TgDCX do not affect microtubule binding or curving in Xenopus S3 cells. Deconvolved wide-field images of Xenopus S3 cells expressing EGFP-tubulin (green) and either mCherryFP-TgDCX-R152I_D201R (A, red) or mCherryFP-CvDCX2-I85R_R134D (B, red). [file 12860_2020_249_MOESM2_ESM.pdf]
